# The pro-apoptotic effect of a Terpene-rich *Annona cherimola* leaf extract on leukemic cell lines

**DOI:** 10.1186/s12906-019-2768-1

**Published:** 2019-12-12

**Authors:** Carl Ammoury, Maria Younes, Marianne El Khoury, Mohammad H. Hodroj, Tony Haykal, Peter Nasr, Marilyne Sily, Robin I. Taleb, Rita Sarkis, Rana Khalife, Sandra Rizk

**Affiliations:** 1 Department of Natural Sciences, Lebanese American University, Byblos, Lebanon; 2 Laboratory of Regenerative Hematopoiesis, Swiss Institute for Experimental Cancer Research (ISREC) & Institute of Bioengineering (IBI), School of Life Sciences, Ecole Polytechnique Fédérale de Lausanne (EPFL), Lausanne, Switzerland; 3 Biochemical Engineering Department, UCL, London, UK

**Keywords:** *Annona cherimola*, Acute myeloid leukemia, Apoptosis, Cancer, Terpenes

## Abstract

**Background:**

The edible fruit *Annona cherimola* has previously shown many nutritional and medicinal properties. The current study evaluates the anti-cancer and anti-proliferative properties of *Annona cherimola* ethanolic leaf extract (AELE) on Acute Myeloid Leukemia (AML) cell lines cultured in vitro (Monomac-1 and KG-1).

**Methods:**

The anti-proliferative effect of *A. cherimola* ethanolic leaf extract was evaluated via cell viability assay. Its pro-apoptotic effect was assessed through Cell Death ELISA and dual Annexin V/PI staining. To further investigate the molecular mechanism by which the extract promoted apoptosis and inhibited the proliferation of the AML cells used, apoptotic protein expression was determined through western blots. Extract composition was elucidated by Gas Chromatography-Mass Spectrometry (GC-MS).

**Results:**

Our results showed that the treatment with *A. cherimola* ethanolic leaf extract exhibited an inhibitory effect on the proliferation of both cancer cell lines used in a dose- and time-dependent manner, with no toxic effects on normal mononuclear cells (MNCs) isolated from human bone marrow. This effect was mediated by DNA fragmentation and apoptosis, as revealed by Cell Death ELISA and dual Annexin V/PI staining. Western blot analysis revealed a Bax/Bcl2 dependent mechanism of apoptosis, as well as PARP cleavage, confirming the apoptotic results observed previously. These effects may be attributed to the presence of terpenes which constitute a large component of the leafy extract, as revealed via GC-MS.

**Conclusion:**

All the data presented in our study show that the terpene-rich *A. cherimola* ethanolic leaf extract exhibits an anti-proliferative and pro-apoptotic effect on the AML cell lines used.

## Background

Plant-derived compounds have been extensively used in the pharmaceutical industry for the treatment of several human diseases [1–3]. According to the world health organization (WHO), traditional medicine constitutes more than 80% of the total world’s population primary health care needs [4]. Various phytochemicals such as alkaloids, flavonoids, lactones, terpenoids as well as terpenes are known to be key immunomodulators specifically as effective anti-inflammatory and anticancer agents [5–8].

One family of plants that has extensive traditional use is the Annonaceae. *Annona*, which is a genus of flowering plants in this sugar apple family, includes approximately 166 species and is considered the second largest genus in this family. The origin of the generic name is anόn, a Hispaniolan Taίno name for the fruit [9, 10].

Several Annona species were found to exhibit anti-parasitic [11], anti-inflammatory [12], and anti-tumor effects. An extract from *A. glabra, rich in* two diterpenoids displayed a cytotoxic effect on liver cancer cell lines by up-regulating the Bax to Bcl-2 expression ratio [13] and on human leukemia cell lines in vitro [14]*. A. squamosa* chloroform seed extract also showed antitumor and pro-apoptotic effects on murine and human tumor cells through the induction of Reactive Oxygen Species (ROS) [15]. *A. muricata* ethyl acetate leaf extract exhibited a mitochondrial-mediated apoptosis on colon cancer cell lines [16] in vitro, on pancreatic cancer cells [17] in vitro and in vivo, and on breast cancer cell lines [18, 19] by upregulating Bax, p53 and downregulating Bcl-2 proteins. In addition, ethanolic and aqueous extracts from leaves, twigs and roots of *A. muricata* showed a strong anti-proliferative potential and pro-apoptotic effect through G0/G1 cycle arrest [20, 21].

*A. cherimola*, an edible subtropical fruit-bearing species is an evergreen low branched spreading tree [22] that belongs to the Annonaceae family and is commercially cultivated for its edible fruits and traditional uses [23]. Cherimoya, the large green fruit of the tree [24] has an exceptional taste and is reported to have been used as an antioxidant [25, 26] and in phytotherapy for the treatment of several ailments such as stomachache, pancreatic ulcers, skin disease [22, 24]. The various phytochemicals present in *A. cherimola* such as flavonoids, tannins, alkaloids, phytosterols, and terpenoids are traditionally utilized in the treatment of diabetes, nervous disorders and even cancer [25, 27]. Furthermore, annomolin and acetogenins, isolated from *A. cherimola* seed extracts [28], demonstrated a cytotoxic and pro-apoptotic effect in human prostate [29], breast [30], and colon [30] cancer cell lines. Moreover, *A. cherimola* leaves are sold and consumed by people to improve their health, such as in the treatment of hypercholesterolemia in Azores [31]. Other studies on ethanolic leaf extracts revealed an antitumor activity in human larynx epidermoid carcinoma cells in vitro [32].

The current study aims to explore the anti-cancer and anti-proliferative effects of a terpene-rich *A. cherimola* ethanolic leaf extract on acute myeloid leukemia cell lines in vitro.

## Methods

### Isolation and culture of normal mononuclear cells from human bone marrow

Normal mononuclear cells (MNCs)were offered by Prof. Marwan El-Sabban’s Lab at the American university of Beirut (AUB) as a kind gift. The MNCs were obtained originally from bone marrow (BM) aspirate leftovers of healthy patients attending AUB Medical center (AUB-MC). BM aspirates were centrifuged on Ficoll/Hypaque (GE Healthcare Life Sciences, Uppsala, Sweden), a density gradient step to separate MNCs from red blood cells and neutrophils. Then the buffy coat, which is the fraction of the anticoagulated blood containing most of the white blood cells, was aspirated and seeded in petri dishes using Dulbecco’s Modified Eagle’s Medium (DMEM)-low glucose (Sigma, D6046) supplemented with 10% FBS (FBS GibcoTM) and antibiotics (100 U/mL penicillin and 100 μg/mL streptomycin, Lonza) in a humidified incubator at 37 °C and 5% CO_2_. One week later, the cells in suspension were collected as a purified MNCs population and cultured in the same conditions mentioned formerly [33]. DMEM-low glucose complete medium was used in performing cytotoxicity assays on MNCs.

### Cell culture

Two Acute Myeloid Leukemia (AML) cell lines were obtained from American Type Culture Collection: Monomac-1, established from the peripheral blood of a 64-year old AML patient, and KG-1, established from a 59-year old Caucasian male patient. The cells were cultured in RPMI-1640 Sigma-Aldrich (Roswell Park Memorial Institute) media supplemented with 10% fetal bovine serum (FBS Gibco™) and antibiotics (100 μg/mL of streptomycin, and 100 U/mL of penicillin from Pen-Strep Lonza) in a humidified atmosphere containing 5% CO_2_ at 37 °C, and split as previously mentioned by Hodroj et al. [34]

### Plant material

*Annona cherimola* leaves were collected from a tree in Awkar-Lebanon (90 m Above Sea Level), in January 2018, and identified by Dr. Nisrine Machaka-Houri. A voucher specimen was deposited in Beirut Arab University Herbarium (RCED2019–362).

### Preparation of crude leaf extract

Leaves (91.3 g) were grinded, shaken and the extract was then prepared as previously described by Haykal et al [35]. The crude extract was weighed then dissolved in Dimethyl sulfoxide (DMSO) and diluted with RPMI to a final concentration of 8650 μg/ml at 5% DMSO. The stock solution was diluted when needed with RPMI to be applied on cells. DMSO level maximally reached was 0.4% at 692 μg/ml.

### Cell viability assay

Wells were prepared and treated in triplicates with increasing concentrations (173 μg/mL, 346 μg/mL, 519 μg/mL and 692 μg/mL) of AELE with one interference well, for 24 h, 48 h or 72 h. For this purpose, AML cells were counted and seeded in 96-well plates at a density of 3 × 10^5^ cells/mL, and were incubated overnight before treatment. The effect of AELE was assayed at these different timeframes using the MTS cell viability reagent (Promega) according to the Manufacturer’s instructions. Cell proliferation was assessed via spectrophotometry by recording the absorbance at a wavelength of 492 nm, using Varioskan™ LUX multimode microplate reader to detect metabolically active cells. Percentage proliferation was calculated by dividing the absorbance of the treated cells with the average absorbance of the control untreated cells. IC_50_ values were calculated using GraphPad Prism 8.

### Apoptosis detection using cell death detection ELISA

Cells were seeded and incubated overnight at a density of 1 × 10^5^ cells/ml in 24-well plates. Triplicates of wells treated with two increasing concentrations of AELE for 24 h, were prepared and then compared to untreated control cells. A positive control well, treated with 100 μM of etoposide (Abcam), was also included. Cells were extracted and lysed with incubation buffer, using the Cell Death ELISA kit (Roche), before isolation of fragmented cytosolic DNA. The procedure was then completed as previously described by Ghanem et al. [36]

### Apoptosis quantification by Annexin/PI staining

Cells were seeded and incubated overnight at a density of 1 × 10^5^ cells/ml in 24-well plates. After incubation for 24 h with increasing concentrations of AELE, samples were collected, centrifuged at 1500 rpm and 4 °C, resuspended in suspension buffer and stained with Annexin and Propidium Iodine (PI) (Annexin V–fluorescein isothiocyanate [FITC] Apoptosis Detection Kit, Abcam). Samples were immediately analyzed using Accuri C6 flow cytometer.

### Western blot

Cells were seeded and incubated overnight at a density of 3 × 10^5^ cells/ml in 6-well plates, followed by treatment with increasing concentrations of AELE for 24 h. Total proteins were extracted, quantified, separated and transferred to polyvinylidene difluoride (PVDF) membranes,which were then blocked as previously stated by Abou Najem et al [37].

The membranes were incubated with primary antibodies anti-β-actin (Santa Cruz Biotechnology, Dallas, TX, USA), anti-Bax (Elabscience, Houston, TX, USA), anti-Bcl2 (Elabscience, Houston, TX, USA), and anti-cPARP (Abcam, Cambridge, UK), overnight in the fridge, with 2% skimmed dry milk in PBS with 0.05% Tween 20, at the manufacturer’s recommended concentrations: 1/1000 for anti-Bax, anti-Bcl2, anti-cPARP and 1/3000 for anti-actin. After washing, the membranes were incubated with anti-mouse secondary antibody (Bio-Rad, Hercules, CA, USA) at the recommended concentration (2:5000) for 1 h at room temperature. Another wash was performed, before imaging using Clarity™ Western ECL Substrate (Abcam, Cambridge, UK) on ChemiDoc machine (BioRad, Hercules, CA, USA). The ImageJ computer program was used to quantify the blot bands, in order to calculate the relative expression of proteins [37].

### Gas chromatography – mass spectrometry

AELE was analyzed via GC-MS as detailed earlier [35], and the peaks were identified from the literature (NIST11 and Wiley9).

### Statistical analysis

All the experiments were carried out in triplicate and each experiment was repeated three times. The error bars are reported as mean ± SEM. Statistical analysis and *p*-values were calculated by t-tests or two-way ANOVA depending on the experiment. Significant differences were reported with * indicating a p-value: 0.01 < *p* < 0.05, ** indicating a p-value: 0.001 < *p* < 0.01, *** indicating a p-value: 0.0001 < *p* < 0.001 and **** indicating a p-value: *p* < 0.0001.

## Results

### The effect of *A. cherimola* ethanolic leaf extracts on cell proliferation

**Fig. 1:**
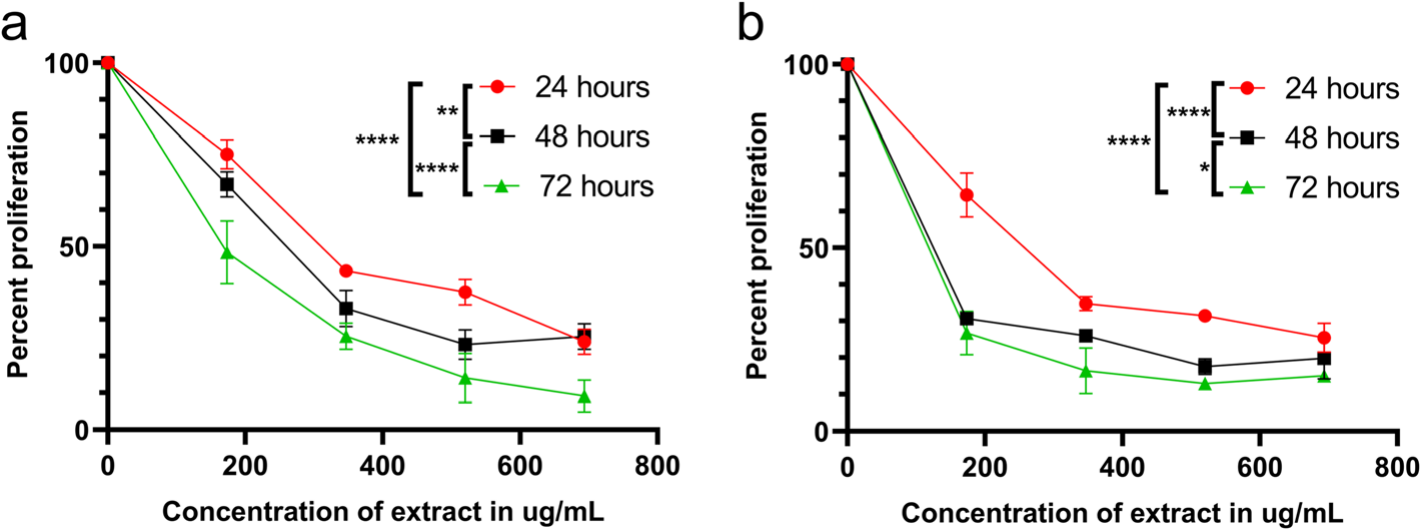
The effect of AELE on cell proliferation using MTS assay. Proliferation of Monomac-1(**a**) and KG-1 (**b**) cells after 24, 48, and 72 h of treatment with increasing concentrations of AELE. The absorbance was measured at 492 nm. A significant dose and time-dependent decrease in proliferation of AML cells was observed upon increasing concentrations of AELE. The IC50 s were reached at 333.4 μg/mL for Monomac-1 and 254.5 μg/mL for KG-1 at 24 h. A time-dependent decrease in the IC_50_ s was observed for both cell lines at 48 and 72 h. (* indicates a *p*-value: 0.01 < *p* < 0.05, ** indicates a p- value: 0.001 < *p* < 0.01, and **** indicates a p-value: *p* < 0.0001)

**Fig. 2:**
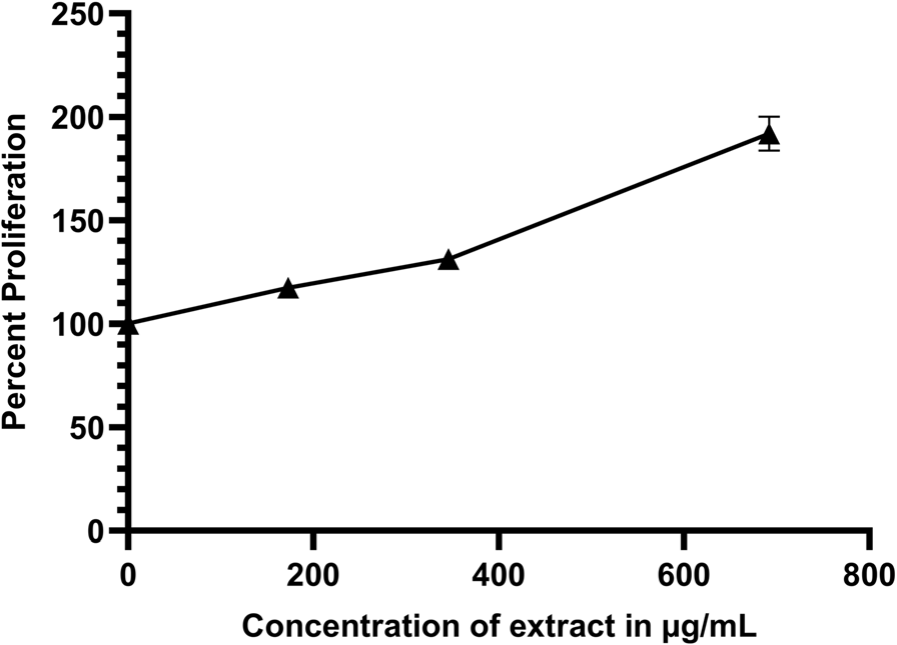
The effect of AELE on MNCs isolated from Human Bone Marrow. AELE showed no inhibitory effect on Mononuclear Cells (MNCs) isolated from Human Bone Marrow

The effect of AELE on Monomac-1 and KG-1 cells was quantified using the cell viability reagent MTS (Promega) via spectrophotometry. The viability was significantly reduced to less than 50% at higher doses. Treatment for longer time had a higher inhibitory effect. The extract exhibited anti-proliferative effects on the two AML cell lines in a dose and time-dependent manner, with a half-maximal inhibitory concentration (IC_50_) of 333.4 μg/mL, 254.1 μg/mL and 168 μg/mL for Monomac-1 (Fig. 1a), 254.5 μg/mL, 34.8 μg/mL and 31.9 μg/mL for KG-1 (Fig. 1b), at 24, 48, and 72 h post-AELE treatment, respectively. The maximum concentration of treatment used (692 μg/ml), exhibited a percentage proliferation of 23.87, 25.37 and 9.10% for Monomac-1 cells, and 25.39, 19.82 and 15.07% for KG-1 cells, at 24, 48 and 72 h after treatment, respectively. AELE showed no inhibitory effect on the viability of normal MNCs from human BM (Fig. 2). This indicated that AELE exhibited selective anti-proliferative effects on all AML cell lines used, which was specific to AML cells, with no toxic effects on normal MNCs. All subsequent experiments were performed 24 h after treating the cells with AELE, in order to elucidate the underlying cellular mechanisms being altered prior to cell death.

### The effect of *A. cherimola* ethanolic leaf extracts on the induction of apoptosis

**Fig. 3:**
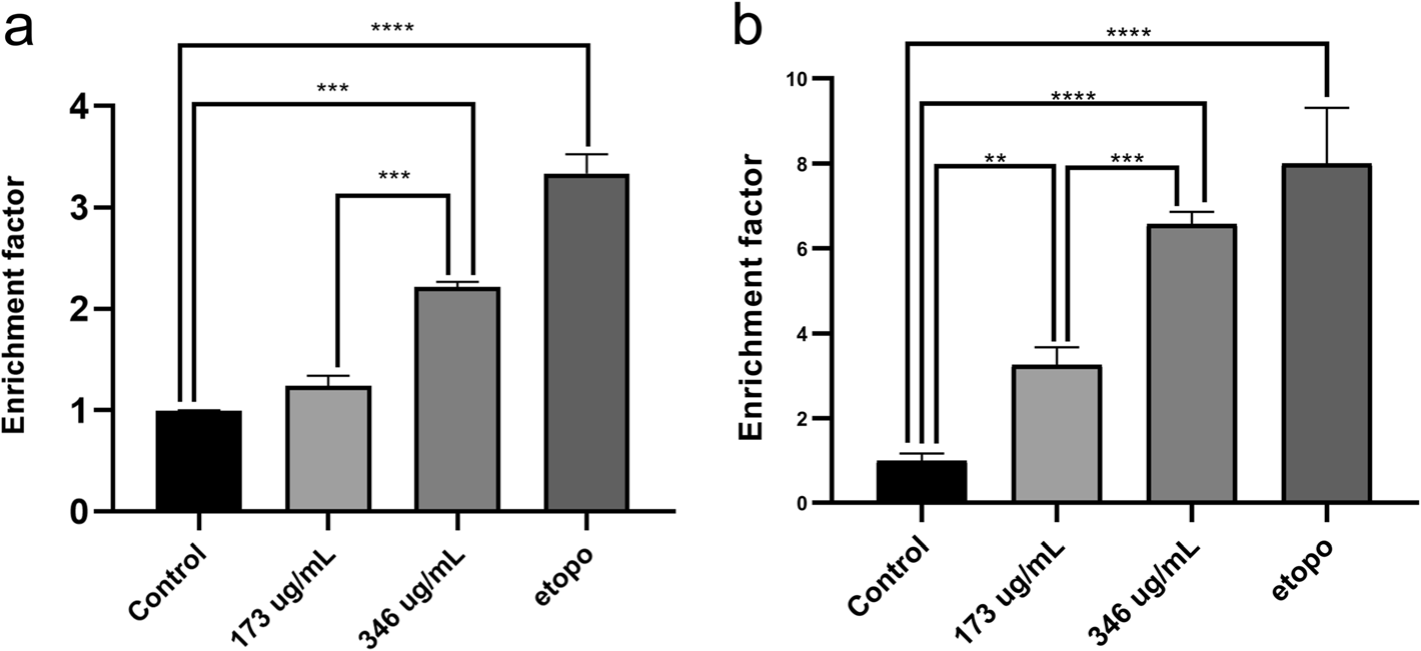
The quantitative effect of AELE on induction of apoptosis using Cell Death ELISA. Cell Death ELISA on Monomac-1 (**a**) and KG-1 (**b**) cells, treated with the two concentrations of AELE closest to the IC50 (173 and 346 μg/mL), as well as a positive control treated with etoposide for 24 h. A significant dose-dependent increase in enrichment factor is noted for AML cells upon treatment with two increasing doses of AELE for 24 h. (** indicates a p- value: 0.001 < *p* < 0.01, *** indicates a p-value: 0.0001 < *p* < 0.001 and **** indicates a p- value: *p* < 0.0001)

After determining the concentrations within which the IC_50_ falls, in Monomac-1 and KG-1 upon treatment with AELE for 24 h, the effect of the extract on induction of apoptosis was quantitatively assessed using Cell Death detection ELISA. In this technique, the enrichment factor was the ratio of the absorbance measured for each drug to that of the untreated controls. The absorbance reflected the quantity of anti-DNA peroxidase, which in turn reflected the level of DNA fragmentation generated by apoptosis. The treatment showed an increase in the enrichment factors at 24 h, which significantly rose from 1.25 to 2.22 for Monomac-1 (Fig. 3a), and from 3.26 to 6.57 for KG-1 (Fig. 3b), at 173 and 346 μg/ml, respectively. These results revealed the ability of the extract to induce apoptosis in Monomac-1 and KG-1 in a dose-dependent manner (*p* < 0.001).

**Fig. 4:**
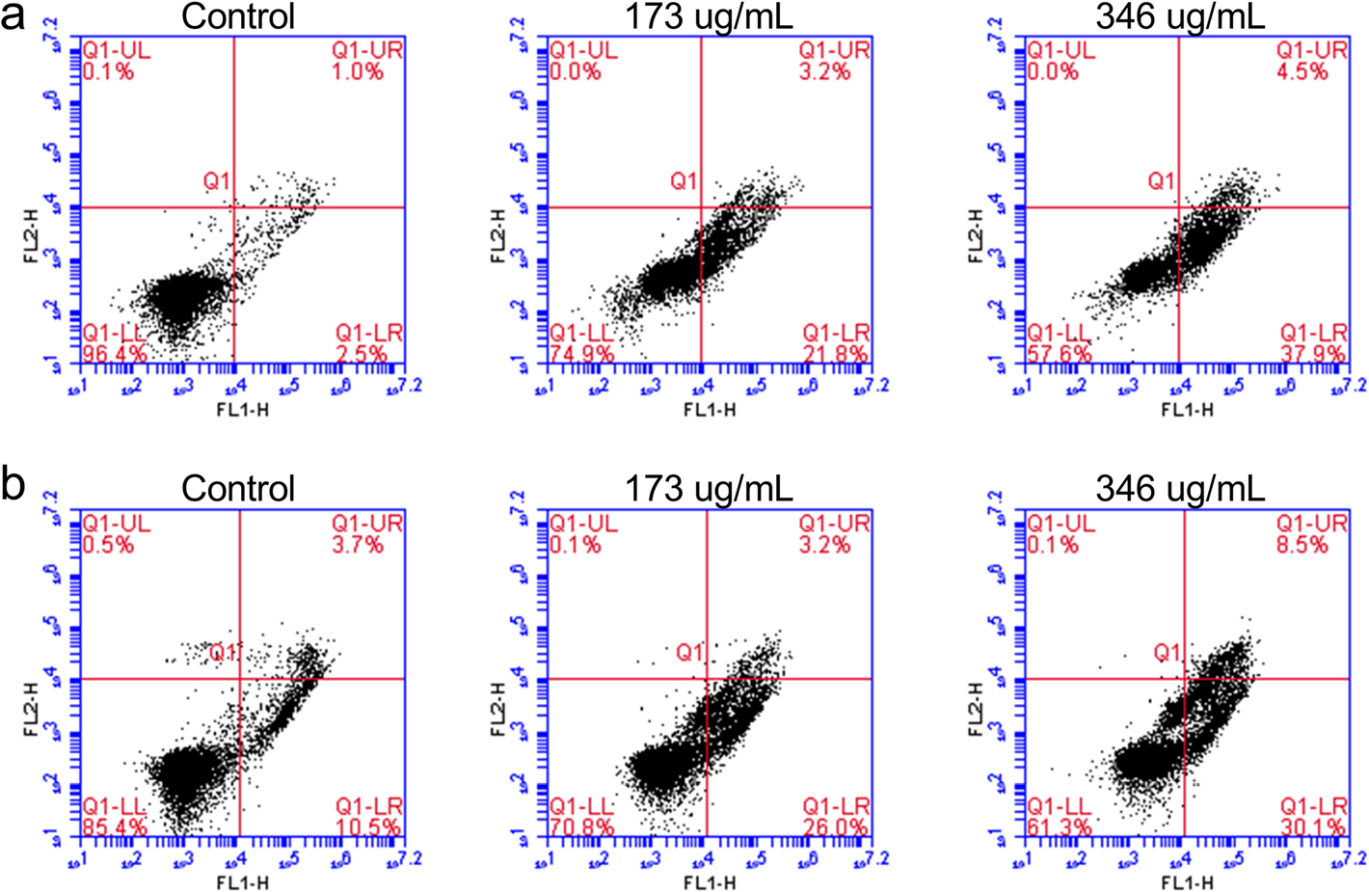
The quantitative assessment of apoptosis induced by AELE using Annexin V/PI. Monomac-1 (**a**) and KG-1 (**b**) were treated with the two concentrations of AELE within which the IC50 falls (173 and 346 μg/mL), followed by staining with Annexin V/PI, and analysis using flow cytometry. A shift from double-negative staining, to Annexin V-positive and PI-negative staining, an early apoptotic marker, upon treatment with AELE was observed. A slight increase in double positive stained cells was also observed

Dual Annexin V/PI staining was used to quantitatively assess apoptosis induction upon various concentrations of AELE treatment. This approach can further determine whether cell death was via apoptotic or necrotic pathways. Cells that stained negative for both Annexin V-FITC and PI (lower left quadrant), were considered normal living cells. Early apoptotic cells were Annexin V-FITC positive and PI negative (lower right quadrant), whereas late apoptotic cells stained positive for both Annexin V-FITC and PI (upper right quadrant). Necrotic cells, on the other hand, exhibit positive staining to PI but negative staining to Annexin V-FITC (upper left quadrant). At 24 h, the percentage of early apoptotic cells increased gradually from 2.5% in untreated Monomac-1 cells to 21.8 and 37.9% at 173 and 346 μg/ml, respectively (before and after the IC_50_) (Fig. 4a).

A similar pattern to the one seen in Monomac-1 cells was observed in KG-1 cells whereby the percentage of early apoptotic cells at 24 h reached 26 and 30.1% at 173 and 346 μg/ml, respectively, compared to the control (10.5%) (Fig. 4b). These results indicated that AELE induced apoptosis in Monomac-1 and KG-1 cells.

### The effect of *A. cherimola* ethanolic leaf extracts on the pro-apoptotic and anti-proliferative pathways

**Fig. 5:**
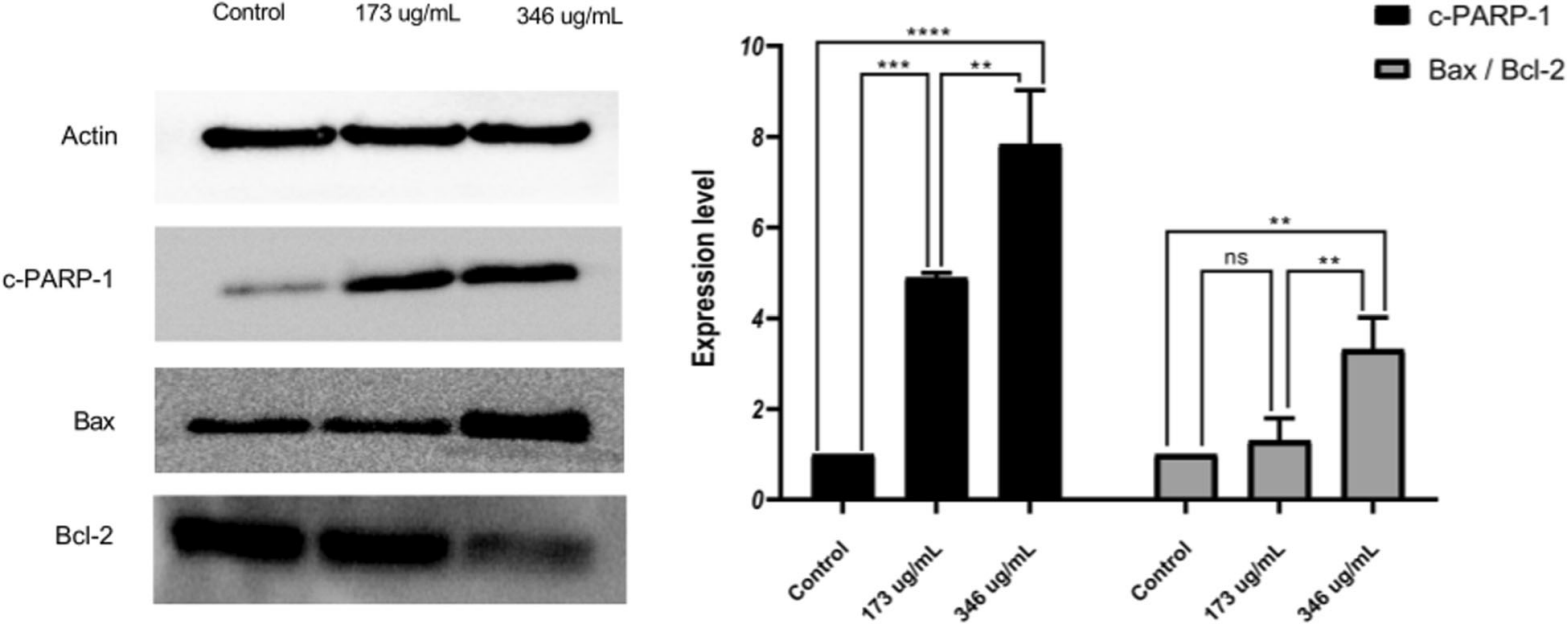
The effect of AELE on the expression of pro- and anti-apoptotic proteins. Western blot analysis and quantification of apoptosis-regulating proteins in Monomac-1 cells treated with AELE for 24 h. Significant upregulation of cleaved PARP-1, and Bax/Bcl-2 ratio was observed between Monomac-1 control cells and cells treated with 173 μg/ml or 346 μg/ml of AELE for 24 h. Representative blots from three different experiments were cropped and are shown in the figure. The full-length blots are reported in the Additional file 1. (** indicates a p-value: 0.001 < *p* < 0.01, *** indicates a p-value: 0.0001 < *p* < 0.001 and **** indicates a *p*-value: *p* < 0.0001)

Since AELE exhibited similar pro-apoptotic effects on both cell lines used, we then focused on Monomac-1 cells to identify the pathway by which AELE promoted apoptosis; the expression of certain proteins related to different pathways was determined using western blot analysis. The cells were treated for 24 h at concentrations closest to the half-maximal inhibitory concentration IC_50_ (173 μg/ml and 346 μg/ml). Beta-actin was used as a housekeeping protein. The pro-apoptotic effect of *A. cherimola* was assessed by measuring the expression of cleaved poly (ADP-ribose) polymerase (PARP), Bax and Bcl-2. Cleaved PARP showed a significant upregulation upon the treatment with increasing concentrations. Moreover, the increase in the Bax to Bcl2 ratio revealed that the pathway by which cells were undergoing apoptosis was Bax/Bcl-2 dependent. These results confirm that apoptosis is triggered upon increasing doses of AELE (Fig. 5).

### Extract composition elucidation by GC-MS

**Fig. 6:**
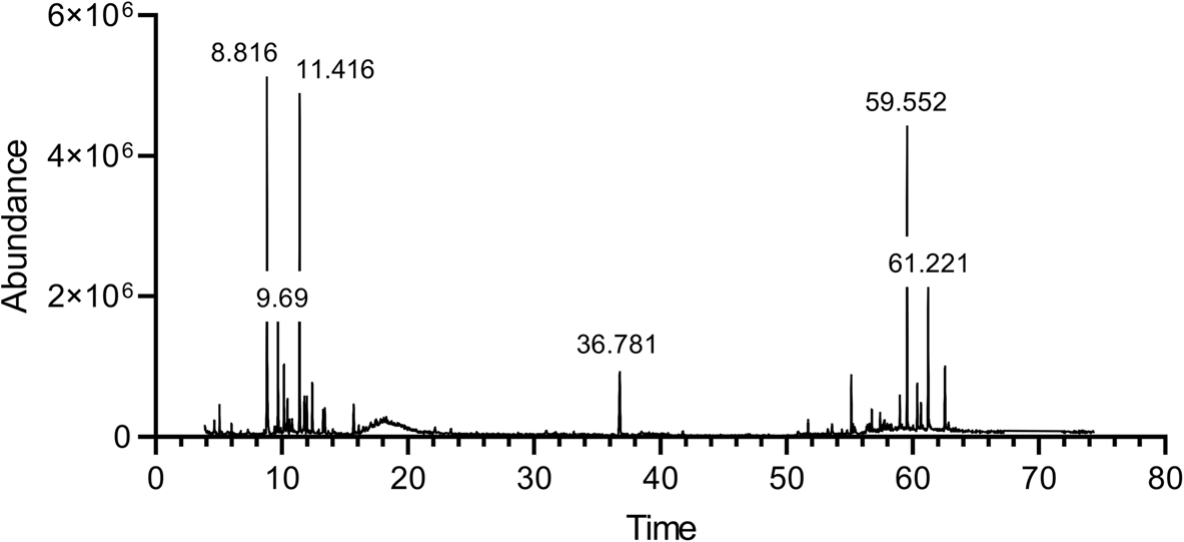
Extract composition elucidation by GC-MS analysis

Gas Chromatography coupled to Mass Spectrometry was performed in order to determine the composition of the extract. The major identified compound was Terpenolene (Retention time 8.8155 min), with an abundance of 16.0619%. The second most abundant detected compound was Germacrene D (Retention time 11.4103 min) with an abundance of 15.2476%, followed by Alpha-Tocopherol (Retention times 59.5517 and 62.5523 min), constituting 15.0038% of the extract. Beta-Sitosterol (Retention time 61.2206 min), was detected with an abundance of 7.0235%. Some other unidentified compounds were detected at retention times 9.69, 10.1644, 10.4387, 13.2736, 13.4107, and 15.6969 min constituting 5.7268, 3.6257, 1.7911, 1.461, 1.4701 and 2.2314% of the extract, respectively (Fig. 6, Table 1).

**Table 1:** The composition of the *A. cherimola* ethanolic leaf extract as elucidated by GC-MS. The major detected compounds were Terpinolene (16.0619%.), Germacrene D (15.2476%), and Alpha-Tocopherol (15.0038%). Other compounds remain unidentified

Peak	RT	Compound	% Extract
1	8.8155	Terpinolene	16.0619
2	9.69	Unidentified A	5.7268
3	10.1644	Unidentified B	3.6257
4	10.4387	Unidentified C	1.7911
5	11.4103	Germacrene D	15.2476
6	11.7876	Gamma-Elemene	1.7086
7	11.9819	Unidentified D	1.4296
8	12.4163	1,3-Cyclopentadiene, 1,2,3,4,5-pentamethyl-	2.725
9	13.2736	Unidentified E	1.461
10	13.4107	Unidentified F	1.4701
11	15.6969	Unidentified G	2.2314
12	36.7813	Phytol	5.9387
13	55.1279	(−)-1,2,3,4-Tetrahydroisoquinoline, 6,7-dimethoxy-2-methyl-1-phenylmethanol	2.8388
14	58.9744	Beta-Tocopherol	1.0881
15	59.5517	Alpha-Tocopherol	11.1711
16	60.3576	Campesterol	2.664
17	60.6491	Stigmasterol	1.3146
18	61.2206	Beta-Sitosterol	7.0235
19	62.5523	Alpha-Tocopherol	3.8327

## Discussion

A correlation between diet and cancer prevention has been demonstrated by the implementation of many plant extracts which exhibited anti-cancerous effects as part of the human diet [10]. Many species classified under the *Annona* genus have shown antitumor effects against several types of cancers, including cervical, breast, prostate, lung, leukemia, colorectal, renal, pancreatic cancers [38]. Many studies have focused on the anti-proliferative effects of *Annona muricata* [16, 18, 20, 21], and *Annona squamosa* [39]. Moreover, most research conducted on *Annona cherimola* leaves has focused on its anti-hyperglycemic [40], and antiprotozoal activity [41], with few studies conducted to assess its anti-proliferative activity. A recent study performed in our laboratory has reported the anti-proliferative effects of *A. cherimola* seed extract via activation of both intrinsic and extrinsic pro-apoptotic pathways in AML cells [35].

The aim of this study was to investigate the mechanism of action of AELE in the apoptotic pathways of the AML cell lines used (Monomac-1 and KG-1), whereby the results suggested a dose- and time-dependent anti-proliferative effect within the 24 h treatment, with an IC_50_ of 333.4 μg/mL and 254.5 μg/mL for Monomac-1 and KG-1, respectively, as well as within the 48 h and 72 h treatment, with a significant time-dependent decrease in the IC_50_ value, and no inhibitory effect on normal MNCs from human BM. Interestingly, AELE exhibited positive effects on the proliferation of normal cells, similar to what has been reported in the literature for other anti-carcinogenic plant extracts such as *Angelica sinensis* [42].

According to Najmuddin et al., crude leaf extracts from *Annona muricata* Linn exhibited anti-cancer effects on breast cancer cell lines, with IC_50_ values at 72 h post-treatment, comparable to the ones reported in this study at 24 h post-AELE treatment, thus suggesting the effectiveness of AELE [18].

All experiments showed that AELE exhibited a dose-dependent increase in apoptosis in the two AML cell lines used. These findings were supported by an increase in DNA fragmentation, as well as the double positive Annexin V/PI staining, indicating the translocation of phosphatidylserine moieties to the outer surface of cell membrane which is a hallmark of apoptosis.

After assessing the anti-proliferative and pro-apoptotic effects of AELE, we moved to decipher the underlying molecular mechanism by which apoptosis was triggered. The results obtained revealed that AELE induces apoptosis through a Bax/Bcl2 dependent mechanism, in concordance with previous studies performed on *Annona muricata* leaves. Dinardo et al. suggested the effectiveness of a selective Bcl2-inhibitor, venetoclax in treating relapsed and refractory AML patients [43]. On the other hand, Reyna et al. developed a pharmacologically optimized Bax activator called BTSA1, which suppressed human AML xenografts, overcoming apoptosis resistance, thus suggesting that direct Bax activation is a possible treatment strategy in AML [44]. Movement of Bax from the cytosol to the mitochondria, through the Bax pores at the mitochondrial membrane, is critical in triggering DNA damage-mediated apoptosis [45, 46], which was observed through the dose-dependent increase in DNA fragmentation detected in cell death Elisa**.** Hence, upregulation of the pro-apoptotic protein Bax detected at 346 μg/ml (at 24 h), accompanied by the downregulation of the anti-apoptotic protein Bcl2, is critical in disrupting the mitochondrial membrane potential, a hallmark of apoptosis. The effect of adding Bax/Bcl2 inhibitors was not further explored since the efficacy of available inhibitors is still controversial [47].

Furthermore, the routine repair of DNA damage is normally controlled by PARP, which adds poly (ADP ribose) polymers in response to a variety of cellular stresses [48]. The increase in PARP cleavage, that was observed upon AELE treatment, will lead to its inactivation, coinciding with its inability to repair DNA damage. This is in line with the dose-dependent increase in DNA fragmentation observed in Cell Death ELISA, further confirming that the cytotoxicity of AELE is indeed apoptosis-triggered.

Upon analysis of the composition of the extract, Terpinolene was found to be the major compound. Terpinolene is one of the most abundant monoterpenes, which is known for its sedative [8]**,** antifungal [49]**,** anticancer, antioxidant [6], apoptotic [50] activities, as well anti-inflammatory and anti-nociceptive activities in association with diclofenac [7]. Interestingly, terpinolene, which is a main constituent of the essential oil of *Protium heptaphyllum*, exhibited an anti-mutagenic activity, suggesting its potential use as a chemo-preventive agent for cancer [51]. α-Pinene, another bicyclic monoterpene, was also found to induce cell cycle arrest in mice Xenograft models, and promote apoptosis in human prostate cancer [52].

The second most common compound in the extract was the sesquiterpene Germacrene D. This compound was previously identified by Bomfim et al., who reported the presence of various sesquiterpenes in the essential oil extracted from *Annona vepretorum* leaves. This extract exhibited in vitro antitumor effects in B16-F10 (mouse melanoma), HL-60 (human promyelocytic leukemia), K562 (human CML), and HepG2 (human hepatocellular carcinoma) cells, as well as in vivo activity [53]. According to Shakeri et al., germacrene D was also found to be the most abundant component in *Nepeta ucrainica* L. spp. *kopetdaghensis,* which was found to be cytotoxic in human ovarian carcinoma A2780 and human breast adenocarcinoma MCF-1 cell lines in vitro [54]. Furthermore, terpenes are the second most common abundant components of *Decatropis bicolor* leaf extracts, which triggered apoptosis in MDA-MB-231 breast cancer cell line, through a Bax/Bcl2 dependent mechanism, translated by a dose-dependent upregulation of Bax, and downregulation of Bcl2 [55]**,** similar to what was observed in our study.

A third major component in AELE was Alpha-tocopherol, an isoform of vitamin E. Zulkapli et al. demonstrated its antitumor activity in oral squamous carcinoma cells ORL-48, whereby accumulation of cells at the sub-G0 phase, along with cell shrinkage and apoptotic bodies were reported [56].

Another identified compound in AELE was β-sitosterol. A study by Zhao et al. reported its ability to inhibit cell growth and trigger apoptosis in SGC-7901 human stomach cancer cells in vitro, in a Bax/Bcl2 and caspase dependent manner [57]. Similar findings were observed on U937 AML cells, involving caspase 3 activation, and an increase in the Bax/Bcl2 ratio [58]. Other studies suggest the anti-inflammatory capacity of β-sitosterol [59], as well as its antihyperglycemic and insulin-releasing activities [60]. Other compounds in AELE remain unidentified and require further investigation. A study performed by Díaz-de-Cerio E. et al reports the presence of polar compounds in *Annona cherimola* leaves using a combined approach of MS and NMR techniques, as well as amino acids, carbohydrates, organic acids, phenolic acids and derivatives, cholines, flavonoids and phenylpropanoids [61].

## Conclusions

In conclusion, *Annona cherimola* ethanolic leaf extracts showed a clear pro-apoptotic effect on Acute Myeloid Leukemia cell lines in vitro. The apoptotic activity of this compound was confirmed through the upregulation of Bax, downregulation of Bcl2, and cleavage of PARP. Chemical analysis of the extract showed that it is also rich in terpenes in addition to other compounds with antioxidant, sedative, anti-inflammatory and antibacterial properties. Further investigations are required to study the effects of the unidentified compounds in the extract, and to confirm the anti-tumor effect of the extract in vivo.

## Additional file

**Additional file 1 **The proapoptotic effect of a Terpene-Rich *annona cherimola* leaf extract on leukemic cell lines.

## Data Availability

Data sharing is not applicable to this article as no datasets were generated or analyzed during the current study. The full length blots have been submitted as supplementary material.

## Abbreviations

AELE: *Annona cherimola* ethanolic leaf extract
AML: Acute Myeloid Leukemia
ANOVA: Analysis of Variance
BM: Bone Marrow
DMEM: Dulbecco’s Modified Eagle Medium
DMSO: Dimethyl Sulfoxide
FBS: Fetal Bovine Serum
GC-MS: Gas Chromatography-Mass Spectrometry
IC50: Half-maximal inhibitory concentration
MNC: Mononuclear Cells
PARP: Poly (ADP-ribose) polymerase
PI: Propidium Iodine
PVDF: Polyvinylidene Difluoride
ROS: Reactive Oxygen Species
RPMI: Roswell Park Memorial Institute
SDS-PAGE: Sodium dodecyl sulfate-Polyacrylamide Gel Electrophoresis
SEM: Standard Error of the mean
WHO: World Health Organization
