# Supplementary material for: The pro-apoptotic effect of a Terpene-rich Annona cherimola leaf extract on leukemic cell lines
Source: BMC Complement Altern Med. 2019 Dec 12;19:365. doi: 10.1186/s12906-019-2768-1 (PMC6909458; doi:10.1186/s12906-019-2768-1)

SUPPLEMENTARY INFORMATION

The proapoptotic effect of a Terpene-rich Annona cherimola leaf extract on leukemic cell lines

Carl Ammoury, Maria Younes, Marianne El-Khoury, Mohammad H Hodroj, Tony Haykal, Peter Nasr, Marylyn Sily, Robin I Taleb, Rita Sarkis, Rana Khalife, Sandra Rizk

Full-length blots that are reported in the manuscript

Beta-actin

Control 173ug/mL 346ug/ml

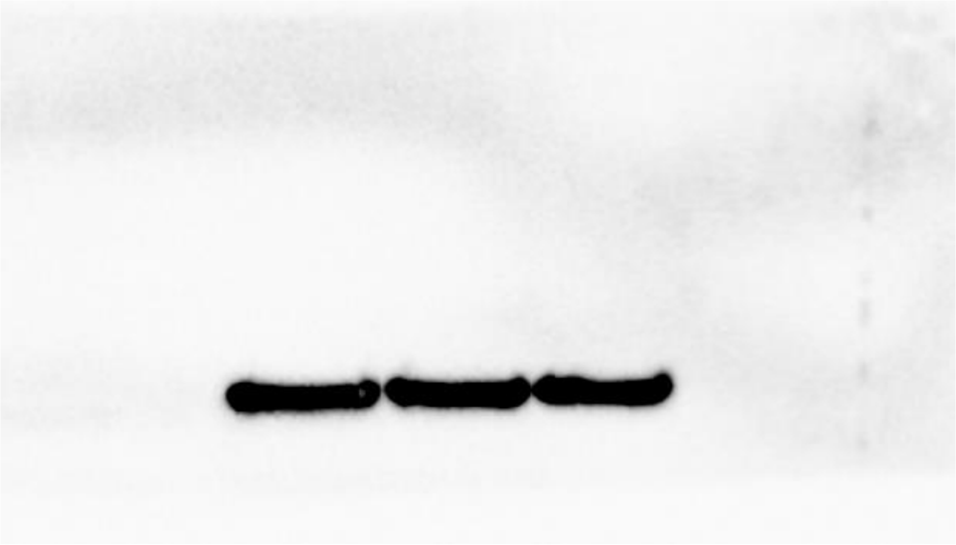

Cleaved PARP

Control 173ug/mL 346ug/ml

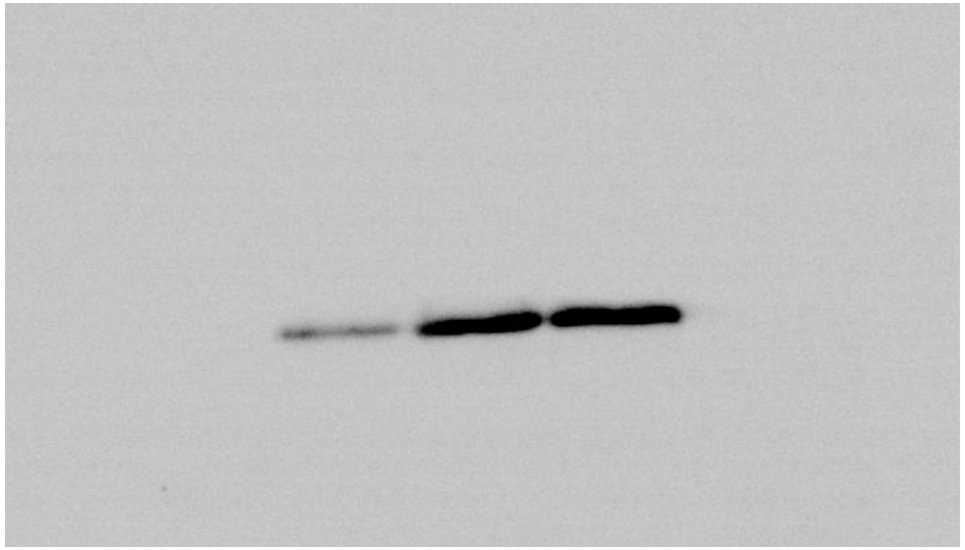

Bax

Control 173ug/mL 346ug/ml

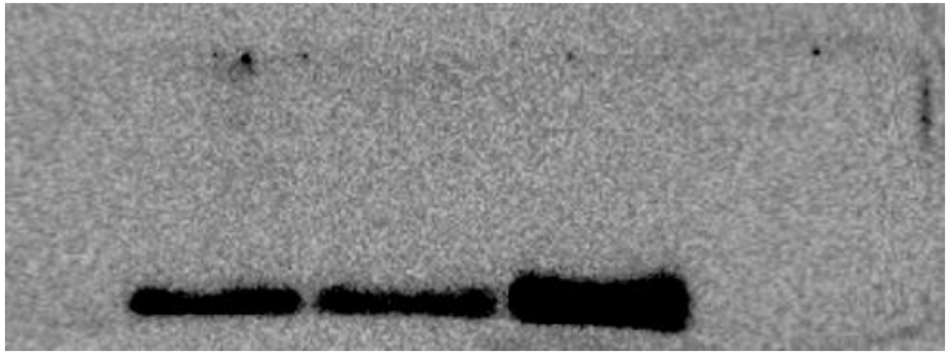

Bcl2

Control 173ug/mL 346ug/ml

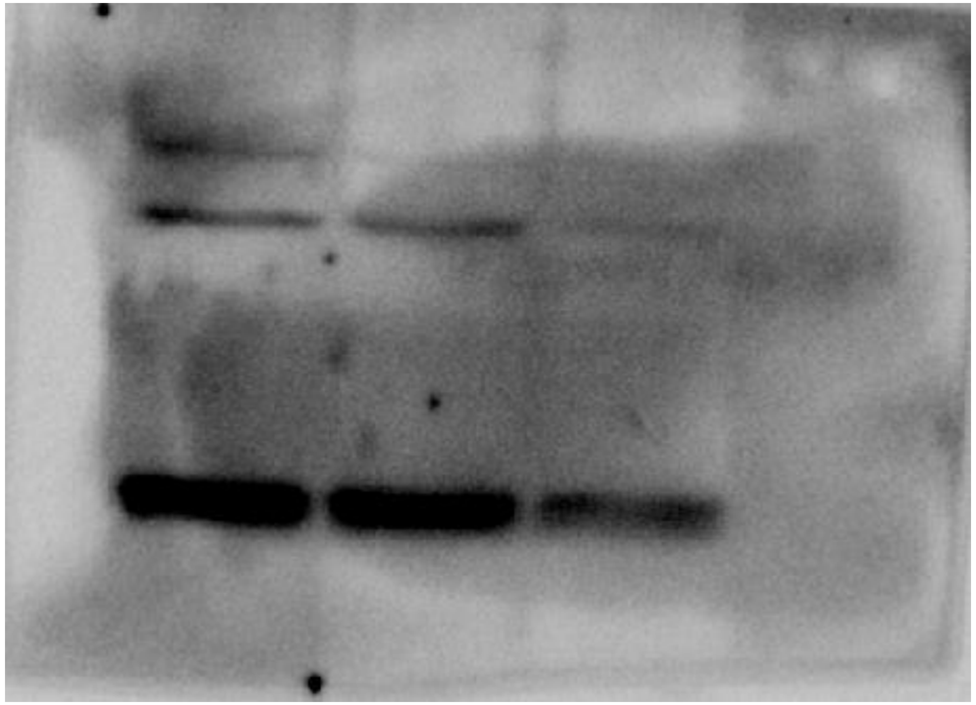

Supplement: Supplementary file 1 — Additional file 1 The proapoptotic effect of a Terpene-Rich annona cherimola leaf extract on leukemic cell lines. [file 12906_2019_2768_MOESM1_ESM.pdf]
